# Serodiagnosis and therapeutic monitoring of New-World tegumentary leishmaniasis using synthetic type-2 glycoinositolphospholipid-based neoglycoproteins

**DOI:** 10.1080/22221751.2022.2114852

**Published:** 2022-09-21

**Authors:** Sayonara M. Viana, Alba L. Montoya, Augusto M. Carvalho, Brunele S. de Mendonça, Susana Portillo, Janet J. Olivas, Nasim H. Karimi, Igor L. Estevao, Uriel Ortega-Rodriguez, Edgar M. Carvalho, Walderez O. Dutra, Rosa A. Maldonaldo, Katja Michael, Camila I. de Oliveira, Igor C. Almeida

**Affiliations:** a Instituto Gonçalo Moniz, Fundação Oswaldo Cruz (FIOCRUZ), Salvador, BA, Brazil.; b Department of Chemistry and Biochemistry, Border Biomedical Research Center, The University of Texas at El Paso, El Paso, Texas, U.S.A.; c Department of Biological Sciences, Border Biomedical Research Center, The University of Texas at El Paso, El Paso, Texas, U.S.A.; d Instituto Nacional de Ciência e Tecnologia de Doenças Tropicais, Salvador, BA, Brazil.; e Departamento de Morfologia, Instituto de Ciências Biológicas, Universidade Federal de Minas Gerais (UFMG), Belo Horizonte, MG, Brazil

**Keywords:** Leishmania braziliensis, tegumentary leishmaniasis, diagnostic and prognostic biomarkers, α-Gal neoglycoproteins, anti-α-Gal antibodies

## Abstract

American tegumentary leishmaniasis (TL) caused by *Leishmania braziliensis* is characterized by a spectrum of clinical presentations, ranging from localized cutaneous ulcers (CL), mucosal (ML), or disseminated (DL) disease, to a subclinical (SC) asymptomatic form. Current diagnosis based on parasite culture and/or microscopy lacks sensitivity and specificity. Previous studies showed that patients with CL and ML have very high levels of *Leishmania*-specific anti-α-Gal antibodies. However, the native parasite α-Gal glycotope(s) is(are) still elusive, thus they have not yet been explored for a more accurate TL diagnosis. Using a chemiluminescent immunoassay, we evaluated the seroreactivity of TL patients across its clinical spectrum, and of endemic (EC) and nonendemic healthy controls (NEC) against three synthetic neoglycoproteins (NGP29b, NGP30b, and NGP28b), respectively comprising the *L. major-*derived type-2 glycoinositolphospholipid (GIPL)-1 (Gal*f*β1,3Manα), GIPL-2 (Galα1,3Gal*f*β1,3Manα), and GIPL-3 (Galα1,6Galα1,3Gal*f*β) glycotopes. Contrary to NGP29b and NGP30b, NGP28b exhibited high sensitivity and specificity to a CL serum pool. More importantly, NGP28b reacted strongly and specifically with individual sera from distinct clinical forms of TL, especially with SC sera, with 94% sensitivity and 97% specificity, by post-two-graph receiver-operating characteristic curve analysis. Contrary to NGP29b, NGP28b showed low cross-reactivity with Chagas disease and control (NEC/EC) sera. Additionally, seroreactivity of CL patients against NGP28b was significantly decreased after successful chemotherapy, indicating that *L. braziliensis*-specific anti-α-Gal antibodies may serve as an early biomarker of cure in CL. Our data also points towards the applicability of *L. major* type-2 GIPL-3-derived Galα1,6Galα1,3Gal*f*β glycotope for the serological diagnosis of American TL, particularly of the subclinical form.

## Introduction

*Leishmania braziliensis* is the leading etiologic agent of tegumentary leishmaniasis (TL) in Brazil [1]. Cutaneous leishmaniasis (CL) is the most prevalent clinical manifestation of TL, with a single or few localized lesions on the skin, from where parasites can metastasize to mucosal sites [2]. Both mucosal (ML) and disseminated leishmaniasis (DL) are metastatic forms also mainly caused by *L. braziliensis* and characterized by high inflammation [3,4]. While ML comprises aggressive lesions with tissue damage, mostly in the oral mucosa, DL is characterized by an elevated number of lesions (>100) ranging from acneiform to ulcerated [5,6]. Moreover, up to 25% of DL patients have ML [7] and DL is associated with a high rate of chemotherapy failure [8].

Pentavalent antimonials are still the first-line drugs against all forms of leishmaniasis but antimonial-based chemotherapy presents high toxicity and adverse events, and in some cases, repeated cycles of treatment are required [9,10]. Therefore, an early and effective diagnosis is essential to ensure a successful response to treatment while avoiding unnecessary drug exposure. The current diagnosis of TL involves clinical and epidemiological data as well as laboratory techniques for the direct or indirect demonstration of the parasite. Parasitological diagnosis through the cultivation of biopsy material or PCR amplification of parasite DNA is highly specific, but sensitivity depends on the non-homogeneous tissue distribution of parasites [11,12]. Among indirect tests based on the host immune response, the enzyme-linked immunosorbent assay (ELISA) is the most commonly used for the serological diagnosis of leishmaniasis. The ELISA sensitivity can vary according to the technique employed and the target antigen(s), and several immunodominant antigens have been identified [1,13–15].

The cell surface of *Leishmania* spp. is abundantly covered with various glycoconjugates such as lipophosphoglycans (LPGs), proteophosphoglycans (PPGs), and glycoinositolphospholipids (GIPLs) [16–18]. In *L. major*, type-2 GIPL-1, -2, and -3 are capped with a terminal nonreducing β-galactofuranosyl (β-Gal*f*) (GIPL-1) or α-galactopyranosyl (α-Gal*p*) (GIPL-2 and -3) residue that is conserved throughout the parasite’s life cycle. Accordingly, anti-α-Gal antibodies have been vastly reported in people infected with kinetoplastids such as certain *Leishmania* spp. and *T. cruzi* [19–29]. These anti-α-Gal antibodies are elicited against parasite-specific molecules and, therefore, have much higher sensitivity and specificity against the pathogen (certain *Leishmania* spp. or *T. cruzi*) than those of healthy subjects anti-α-Gal antibodies directed against enterobacteria [23,25,26,28,30,31].

Using a highly sensitive chemiluminescent ELISA (cELISA), a recent study demonstrated that *L. major*-infected CL patients exhibit high titers of anti-α-Gal antibodies to a commercially available α-Gal-containing neoglycoprotein (NGP, Galα1,3Galβ1,4GlcNAcβ-bovine serum albumin (BSA)) [22]. More recently, using the exact native primary α-Gal glycotope linked to the remaining one or two sugar residues of *L. major* type-2 GIPL-2, Montoya *et al.* [24] showed that NGP27b (Galpα1,3Gal*f*β) and NGP30b (Galpα1,3Gal*f*β1,3Manα), employed in tandem, distinguished with 100% specificity *L. major*-caused CL infection from non-CL heterologous diseases and *L. tropica*-caused CL.

In the present work, we expand these findings to similarly produced synthetic *L. major* type-2 GIPL-based NGPs applied to the serological diagnosis of patients across the TL spectrum caused by *L. braziliensis*. We show that sera from TL patients in the active phase of the disease strongly react with Gal*f*β1,3Manα-BSA (NGP29b) [32], Gal*p*α1,3Gal*f*β1,3Manα-BSA (NGP30b), and Gal*p*1,6Gal*p*α1,3Gal*f*β-BSA (NGP28b) [24], respectively comprising the native *L. major* type-2 GIPL-1, -2, and -3 glycotopes. However, NGP28b exhibits higher sensitivity and specificity than do NGP29b and NGP30b. Furthermore, NGP28b shows significant discriminatory potential for monitoring cured CL from active disease cases, as well as asymptomatic or subclinical (SC) *L. braziliensis-*infected patients. This study furthers our knowledge on the applicability of *Leishmania*-specific anti-α-Gal antibodies as diagnostic tools and potential biomarkers (BMKs) of chemotherapeutic outcomes in cutaneous leishmaniasis caused by *L. braziliensis*.

## Material and methods

### Ethics Statement

This research was conducted with the approval of the Ethical Committee of the Hospital Prof. Edgard Santos (Salvador, Bahia, Brazil; approval number 240/2009), and Comissão Nacional de Ética em Pesquisa (Brazilian National Ethics Committee, Brazil). Informed consent was obtained from each participant.

### Serum and plasma samples

Sera were randomly selected from a bank of serum samples from clinically and laboratory-confirmed cases of TL identified at the Health Post of Corte de Pedra, Bahia, Brazil, a reference center for diagnosis and treatment of leishmaniasis. Plasma samples of SC patients were collected from 2011 to 2015, whereas sera of CL and ML, and plasma from DL patients were collected from 2015 to 2019. Epidemiological and clinical characteristics for patients with CL, ML, DL, or SC forms of TL are described in Table 1. CL and ML active disease were characterized by the presence of one or more ulcerative lesion(s) on the skin site(s), or in the nasal mucosa, respectively, according to diagnosis guidelines [33]. Patients with DL exhibited ten or more acneiform, papular, and ulcerated lesions in at least two different parts of the body [7,34]. Laboratory confirmation of the diagnosis was based on the detection of *L. braziliensis* DNA using polymerase chain reaction (PCR) [35], or by histopathology showing amastigote forms in the tissues collected from lesions [33,34]. Individuals with SC infection were defined as household contacts from CL patients with a positive leishmanin (Montenegro) skin test (LST) without clinical manifestations of CL. Endemic controls (EC) (n = 15) consisted of household contacts of CL patients without clinical manifestations of CL, a negative LST and no production of interferon-γ in vitro [36]. These EC individuals were not screened for any other endemic infection(s) in the region at the time of sample collection. The LST was performed with soluble leishmanial antigen, as previously described [37]. Briefly, twenty-five micrograms of soluble *Leishmania* antigen (SLA) was injected intradermally on the ventral face of the forearm. The test was considered positive when the induration was ≥5 mm after 48 h. The Brazilian Ministry of Health recommends that patients living in an endemic area for *L. braziliensis* get systemic treatment, considering that they could develop more severe forms of TL [33,38]. Patients with active disease were treated daily with the standard meglumine antimoniate - Sb^v^ (Glucantime, Sanofi-Aventis) therapy (20 mg Sb^v^/kg/day for 20 consecutive days) for CL and ML, and a regimen of 20 mg Sb^v^/kg/day for 30 consecutive days for DL [38]. The percentage of patients cured at 90 days post-treatment is considered the primary outcome for evaluation of intervention efficacy in clinical trials for American CL and ML [39]. For CL and ML patients, sera were obtained both at the time of diagnosis (day 0, active CL/ML) and following clinical confirmation of cure (day 90, cured CL/ML). For patients with other clinical forms of TL (DL and SC), sera were obtained at the time of diagnosis only. Additional sera were obtained from chronic Chagas disease (CD, n = 16) patients, or from healthy nonendemic controls (NECs, n = 18) residents of Salvador, BA, Brazil. NEC individuals showed negative responses to both anti-*Leishmania* serology and LST. The study was approved by the Institutional Review Board at the Medical School, Federal University of Bahia.

**Table 1.** Demographics and clinical characteristics of tegumentary leishmaniasis patients.

Variable	TL form ^a^				*p*^b^
	CL (n = 17)	ML (n = 16)	DL (n = 16)	SC (n = 31)	
Age, years ^c^	32 (12.4)	45 (18)	39 (15)^e^	26 (13.9)	0.02 (CL vs. ML); 0.0002 (ML vs. SC); 0.006 (DL vs. SC)
Number of males (%)	12 (70.6)	7 (43.8)	15 (93.8)	16 (51.6)	-
Number of lesions ^d^	1 (1)	-	20 (13-35) ^e^	-	-
LST area (mm^2^) ^a,d^	204 (170.3−261.4)	266 (147.5−319)^f^	113 (4.8−175.8)	104 (44.0−240.2)	<0.0001 (CL vs. DL); 0.0067 (ML vs.DL); 0.0380 (CL vs. SC); 0.0299 (ML vs. SC)
Healing time (days) ^c^	45.1 (18.6)	59.6 (17.7)	137.8 (61.9) ^e^	-	0.0294 (CL vs. ML); 0.0001 (CL vs. DL); 0.0001 (ML vs. DL)

^a^ Abbreviations: LST, leishmanin (Montenegro) skin test; CL, cutaneous leishmaniasis; ML, mucocutaneous leishmaniasis; DL, disseminated leishmaniasis; SC, subclinical leishmaniasis.

^b^ The Student's *t-*test or Mann Whitney test were used to compare continuous variables and the Fisher's exact test to compare proportions.

^c^ Mean (SD)

^d^ Median (interquartile range, IQR)

^e^ Data missing from one subject.

^f^ Data missing from three subjects.

### Neoglycoproteins (NGPs)

**Figure 1:**
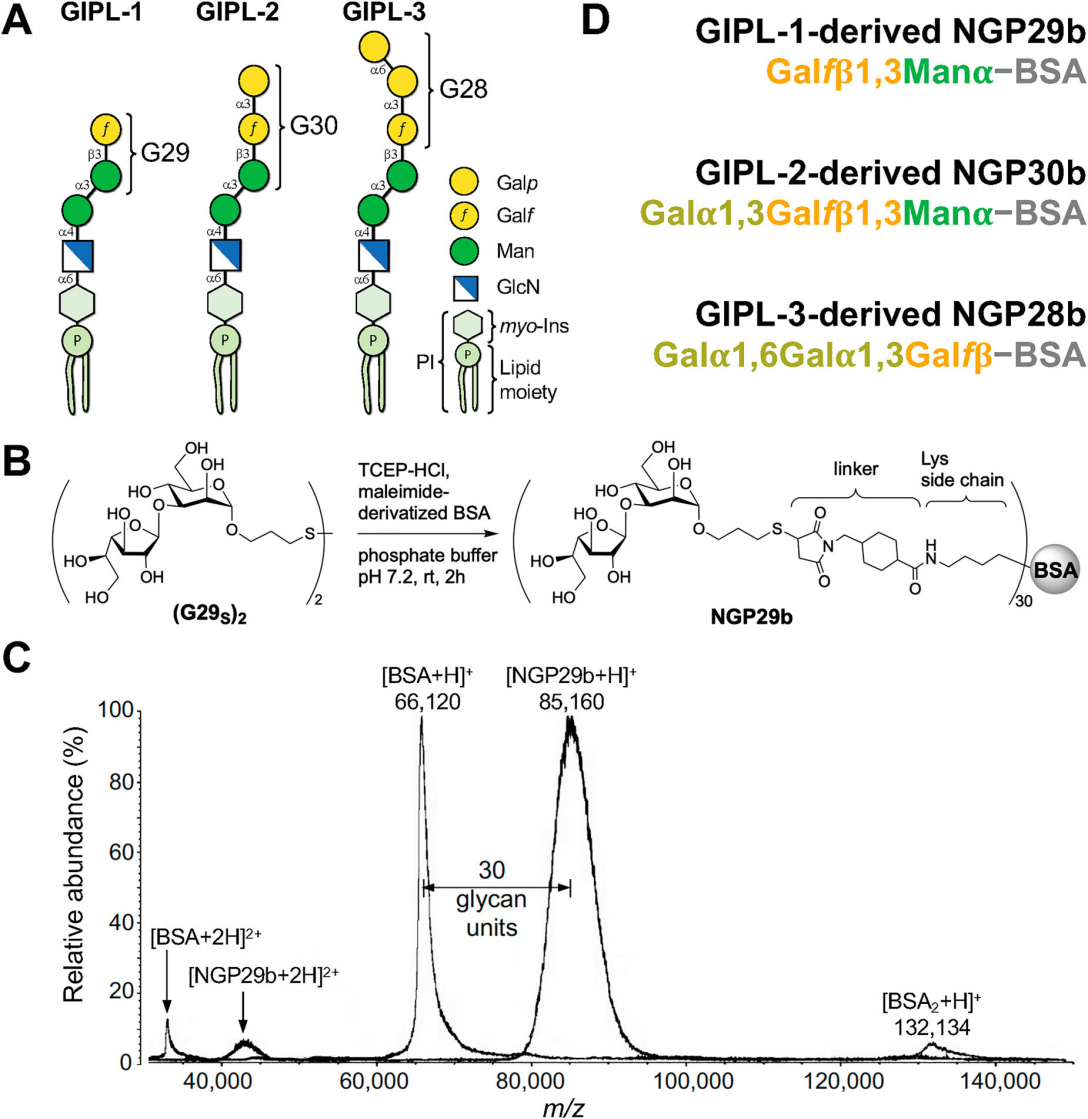
**.** Synthetic neoglycoproteins used in this study. (**A**) Schematic representation of type-2 GIPLs 1-3 of *L. major*. The terminal glycan moiety (G29, G30, or G28) targeted for chemical synthesis in each GIPL is indicated. Gal*p*, galactopyranose; Gal*f*, galactofuranose; Man, mannopyranose; GlcN, glucosamine; *myo*-Ins, *myo*-inositol; P, phosphate; PI, phosphatidylinositol. (**B**) Schematic representation of the synthesis of NGP29b containing the type-2 GIPL-1 terminal, nonreducing glycotope Gal*f*β1,3Manα. TCEP-HCl, Tris (2-carboxyethyl) phosphine hydrochloride; linker, 4-(succinimidomethyl)cyclohexane-1-carboxy group. The same conjugation was used for the synthesis of NGP30b and NGP28b [24]. (**C**) Representative MALDI-TOF-MS spectrum of NGP29b to confirm the covalent conjugation of the glycan units to the carrier protein, as recently described [32]. The same quality-control procedure was used for NGP30b and NGP28b, as previously described [24]. Doubly charged ([BSA+2H]^+2^ and [NGP29b+2H]^+2^) and singly charged ([BSA + H]^+^, [NGP29b + H]^+^, and [BSA_2 _+ H]^+^) ions of BSA and NGP29b are indicated. The number of glycan units (n = 30) covalently attached to the BSA moiety is indicated. *m/z*, mass to charge ratio. (**D**) Composition of the synthetic NGP29b, NGP30b, and NGP28b. For simplicity, the glycan thiopropyl group (at the reducing end), the linker covalently attached to the lysine residue, and the number of glycan units shown in **B**, are not indicated.

The mercaptopropyl glycoside derivative G29_SH_ (Gal*f*β1,3Manα-3-mercaptopropyl) structure was based on the *L. major* type-2 GIPL-1 [16,40] (Figure 1A), and it was synthesized as recently described [32]. The purity of the disulfide forms of G29_SH_ ([G29_S_]_2_) was ∼95%, as assessed by nuclear magnetic resonance spectroscopy NMR spectroscopy (^1^H-NMR [400 MHz, D_2_O, 300 K] and ^13^C-NMR [100 MHz, D_2_O, 300 K]) of the disulfide form of G29_SH_ ([G29_SH_]_2_) [32]. Upon reduction of (G29_SH_)_2_ with tris(2-carboxyethyl)phosphine hydrochloride (TCEP-HCl), the ensuing G29_SH_ was conjugated to commercial maleimide-derivatized BSA (Imject™ Maleimide-Activated BSA, Thermo Fisher Scientific) to give rise to NGP29b (Gal*f*β1,3Manα-BSA), whose purity (∼99%) was evaluated by MALDI-TOF-MS (Figure 1B-D) [32]. The mercaptopropyl glycoside derivatives G30_SH_ (Gal*p*α1,3Gal*f*β1,3Manα-3-mercaptopropyl) and G28_SH_ (Gal*p*1,6Gal*p*α1,3Gal*f*βα-3-mercaptopropyl) structures were based on *L. major* type-2 GIPL-2 and -3, respectively [16,40] (Figure 1A), and were synthesized as previously described [24]. The purity (∼95%) of the disulfide forms of G30_SH_ ([G30_S_]_2_) and G28_SH_ ([G28_S_]_2_) was also estimated by NMR (^1^H-NMR [600 MHz, D_2_O, 300 K] and ^13^C-NMR [150 MHz, D_2_O, 300 K]) [24]. Upon reduction of the mercaptopropyl glycoside derivative disulfide forms ([G30_S_]_2_ and [G28_S_]_2_) with TCEP-HCl, resulting G30_SH_ and G28_SH_ were conjugated to maleimide-derivatized BSA to give rise to NGP30b (Gal*p*α1,3Gal*f*β1,3Manα-BSA) and NGP28b (Galα*p*1,6Gal*p*α1,3Gal*f*β-BSA), respectively, as described [24] (Figure 1D). The purity of both NGPs (∼99%) was assessed by MALDI-TOF-MS [24] (Figure 1C).

### Chemiluminescent enzyme-linked immunosorbent assay (cELISA)

To determine the levels of human IgG antibodies to NGP29b, NGP30b, and NGP28b, these synthetic antigens were cross-titrated at concentrations ranging from 3.13 to 50 ng/well, using pools of 15 sera from each NEC (n = 18), CL (n = 17), or CD (n = 16) serum panel, at 1:400 or 1:800 dilution. White opaque 96-well MaxiSorp Immune Plates (catalog number 436110, Thermo Fisher Scientific) were coated with NGPs overnight (O/N) at 4°C in 100 mM carbonate–bicarbonate buffer, pH 9.6 (CBB). Wells were blocked with 200 μL PBS-1% BSA (PBS-B) for 1 h, at 37°C. Human serum samples, diluted in PBS-B plus 0.05%Tween 20 (PBS-TB) were then added and incubated for 1 h, at 37°C. After washing, plates were sequentially incubated with 50 μL biotinylated goat antihuman IgG (H + L) secondary antibody (1:5,000 dilution in PBS-TB; catalog number 31030, Thermo Fisher Scientific), and 50 μL Pierce High Sensitivity NeutrAvidin-horseradish peroxidase (1:5,000 dilution in PBS-TB; catalog number 31030, Thermo Fisher Scientific). Incubation steps were performed for 30 min at 37°C. Between incubation steps, plates were washed 3x with 250 μL PBS-T. The reaction was developed with SuperSignal ELISA Pico Chemiluminescent Substrate (catalog number 37069, Thermo Fisher Scientific) by diluting the Luminol/Enhancer Solution and Stable Peroxide Solution in CBB, at a 1/1/8 ratio (v/v/v). Luminescence was read in a FilterMax F3 Microplate Reader (Molecular Devices) and values were expressed as relative luminescence units (RLUs). Pools of sera from 15 active CL (aCL), cured CL (cCL) individuals, and 15 NECs were also used as positive (aCL) and negative (cCL and NEC) controls.

Next, we examined the levels of IgG antibodies to NGP29b and NGP28b in individual sera of CL (n = 17), ML (n = 16), and CD (n = 16) patients, plasma of DL (n = 16) and SC (n = 31) patients, and sera of EC (n = 15) and NEC (n = 18) individuals. Each NGP was used at 5 ng/well and each serum (at 1:800 dilution) or plasma (at 1:400 dilution, considering a 1:2 dilution of plasma) sample was tested in technical triplicate. The cELISA protocol was performed exactly as described above. The mean (x̅) RLU value was normalized (as cELISA titer) by dividing it by the cutoff value, calculated as follows: cutoff = x̅ + SD*f*, where x̅ is the mean value of six technical replicates of a pool of sera from NECs in each microplate; SD*f* is the standard deviation (SD) multiplier, calculated based on the number of negative control replicates in each microplate (confidence level [1 – α] of 95% using 6 controls = 2.177), as described [41]. The titer of each cELISA was defined as the ratio of the experimental sample’s average RLU value to the cutoff value. A serum sample was considered positive when its cELISA titer was equal to or higher than 1.000, and negative when the titer was lower than 1.000, as previously described [24].

### Statistical Analysis

The variables in this study were evaluated regarding their distribution with the Kolmogorov–Smirnov test and skewness and kurtosis values obtained using IBM SPSS Statistics 20 software. Once data showed a nonparametric distribution, analyses were performed using Kruskal–Wallis followed by Dunn’s post-test. Cross-titration curves were compared using two-way Anova with main effects only and Dunnett’s multiple comparison test (with individual variances computed for each comparison). Paired comparisons (pre- and posttreatment) were performed using the Wilcoxon Rank Sum test. Statistical significance was set at the conventional 5% level (*p*⩽0.05) and all non-parametric analyses were performed using GraphPad Prism version 9.0 (GraphPad Software, San Diego, CA). Finally, multiple logistic regression models followed by receiver-operating characteristic (ROC) curve analyses were performed on normalized (cELISA titer) values to establish sensitivity, specificity, and other performance parameters obtained from ROC/AUC, two-graph ROC (TG-ROC), *p* values, and likelihood ratio, using GraphPad Prism v. 9.0.

## Results

This cross-sectional, retrospective study evaluated a cohort of patients with distinct clinical forms of TL caused by *L. braziliensis* and individuals with the asymptomatic subclinical (SC) form of the infection. These patients are from the region of Corte de Pedra, Bahia, Brazil, which is a well-studied endemic area for TL [42]. Table 1 shows the demographics and clinical characteristics of the 80 individuals tested across the TL spectrum: the mean age ranged from 26 to 45 years (33.8 ± 16.2), with a predominance of males (62.5%). SC individuals were significantly younger (26 ± 13.9 years old) than ML (45 ± 18) and DL (39 ± 15) patients (*p *= 0.0002, ML vs SC; *p *= 0.006, DL vs SC). Most CL patients (82.4%) exhibited a single lesion, whereas DL patients exhibited a median of 20 ulcers (Table 1). For CL and ML patients, clinical cure was defined by complete healing of the ulcers with reepithelialization without raised borders on day 180 after initiation of treatment [33].

For the evaluation of the presence of *L. braziliensis-*specific anti-α-Gal and anti-β-Gal*f* antibodies in the sera of CL, ML, DL, and SC patients, we first employed three synthetic NGPs containing terminal glycotopes found on *L. major* type-2 GIPL-1, -2, and -3*,* which have previously shown to be highly reactive to sera from American TL caused by *L. braziliensis* [19]. In Figure 1, we show the schematic representations of *L. major* type-2 GIPL-1, -2, and -3, an scheme of the chemical synthesis and a representative quality control (by MALDI-TOF-MS) of NGP28b, and the basic composition of NGP29b, NGP30b, and NGP28b. The inclusion of the terminal, nonreducing β-Gal*f*-bearing NGP29b in this study was based on the premise that β-Gal*f* is a sugar entirely absent in all mammals, thus extremely immunogenic, immunomodulatory, and antigenic to mice and/or humans, hence a potential BMK for American TL [32,43–47].

**Figure 2.**
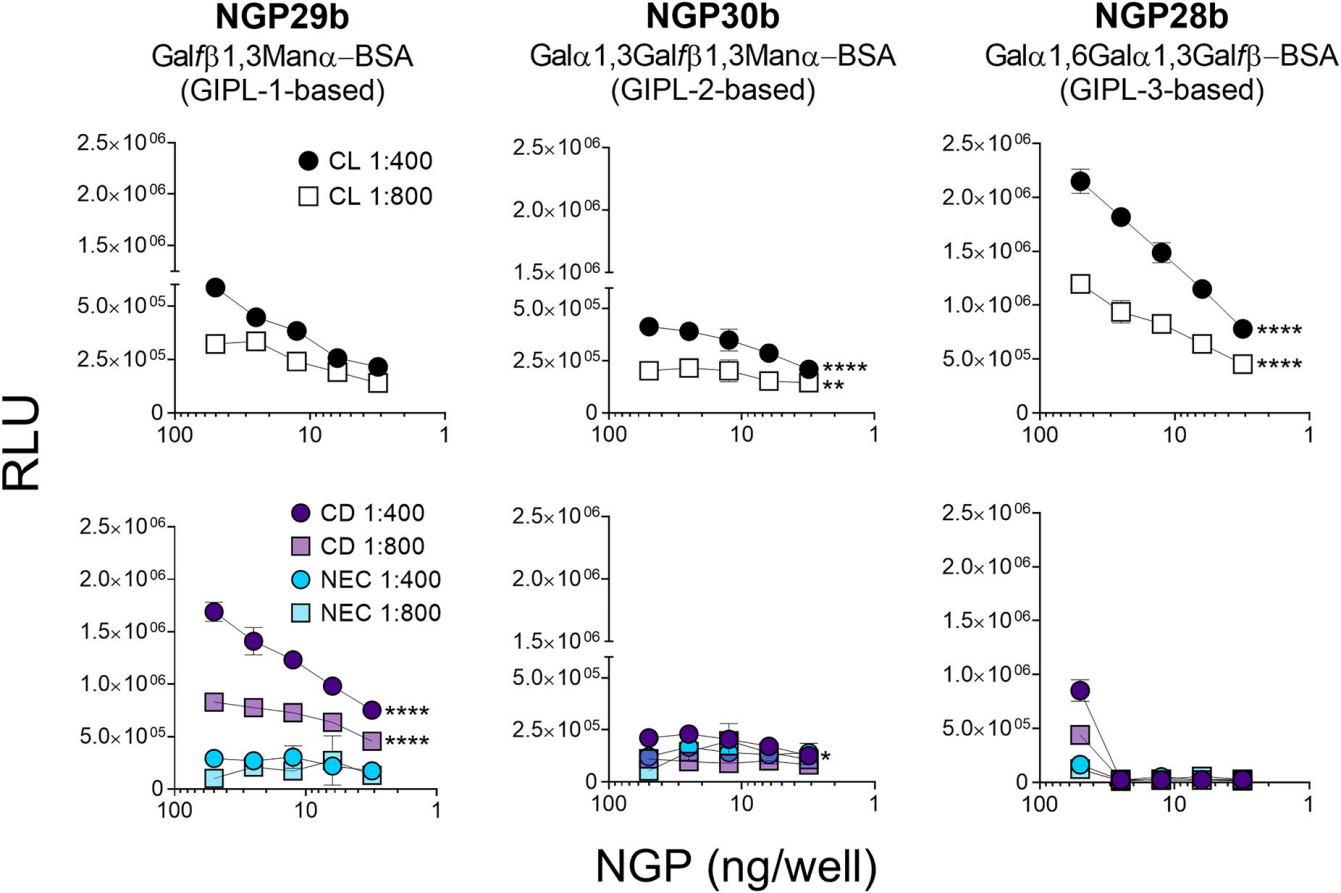
Cross-titration of NGPs with serum pools from CL, Chagas disease, and nonendemic controls. cELISA tests were performed with NGP29b, NGP28b, or NGP30b at concentrations ranging from 50 to 3.13 ng/well, using a pool of sera (n = 15) from patients with active CL, caused by *L. braziliensis*. Pools of sera obtained from patients with chronic Chagas disease (CD) (n = 15), and from nonendemic healthy controls (NEC) (n = 15) were also evaluated. Each point represents the mean of duplicate values of the relative luminescence units (RLU) obtained for each sample and bars indicate SD. Statistical analysis: two-way Anova with main effects only and Dunnett’s multiple comparison test (with individual variances computed for each comparison). The CL and CD curve were compared with the NEC curve, at 1:400 and 1:800 serum pool dilution. *p < 0.05, **p < 0.01,****p < 0.0001; statistically non-significant differences are not shown.

First, we tested the reactivity of sera of CL and CD patients, and NEC individuals to these three synthetic NGPs. Pooled sera (n = 15) from each patient/control panel were tested at 1:400 or 1:800 dilution against a concentration range (50 − 3.1 ng/well) of the three NGPs. The serum pool from CL patients exhibited discrete reactivity to NGP29b (GIPL-1-based), whereas a serum pool from CD patients showed strong reactivity to this NGP, in a dose-dependent manner, at 1:400 and 1:800 dilutions (Figure 2, left panels). NGP30b (GIPL-2-based) was recognized with low RLU values by serum pools from CD patients and NEC individuals, with a significant increase in recognition by the serum pool of CL patients at 1:400 dilution (Figure 2, central panels). Conversely, the serum pool of CL patients exhibited a much stronger reactivity to NGP28b (GIPL-3-based), at both dilutions tested, in a dose–response manner, whereas serum pools of CD patients and NEC individuals exhibited little or no reactivity at concentrations <25 ng/well. Serum pool of NEC individuals showed a very weak or no reactivity to NGP28b at all concentrations tested (Figure 2, right, bottom panel). Taken together, these results indicate that the GIPL-3-based NGP28b is strongly recognized by anti-α-Gal antibodies present in the serum pool from *L. braziliensis*-caused CL, and to a much lesser extent by CD serum pool*.* By contrast, GIPL-1-based NGP29b is more strongly reactive to anti-β-Gal*f* antibodies present in the serum pool of CD patients than those in CL serum pool.

**Figure 3.**
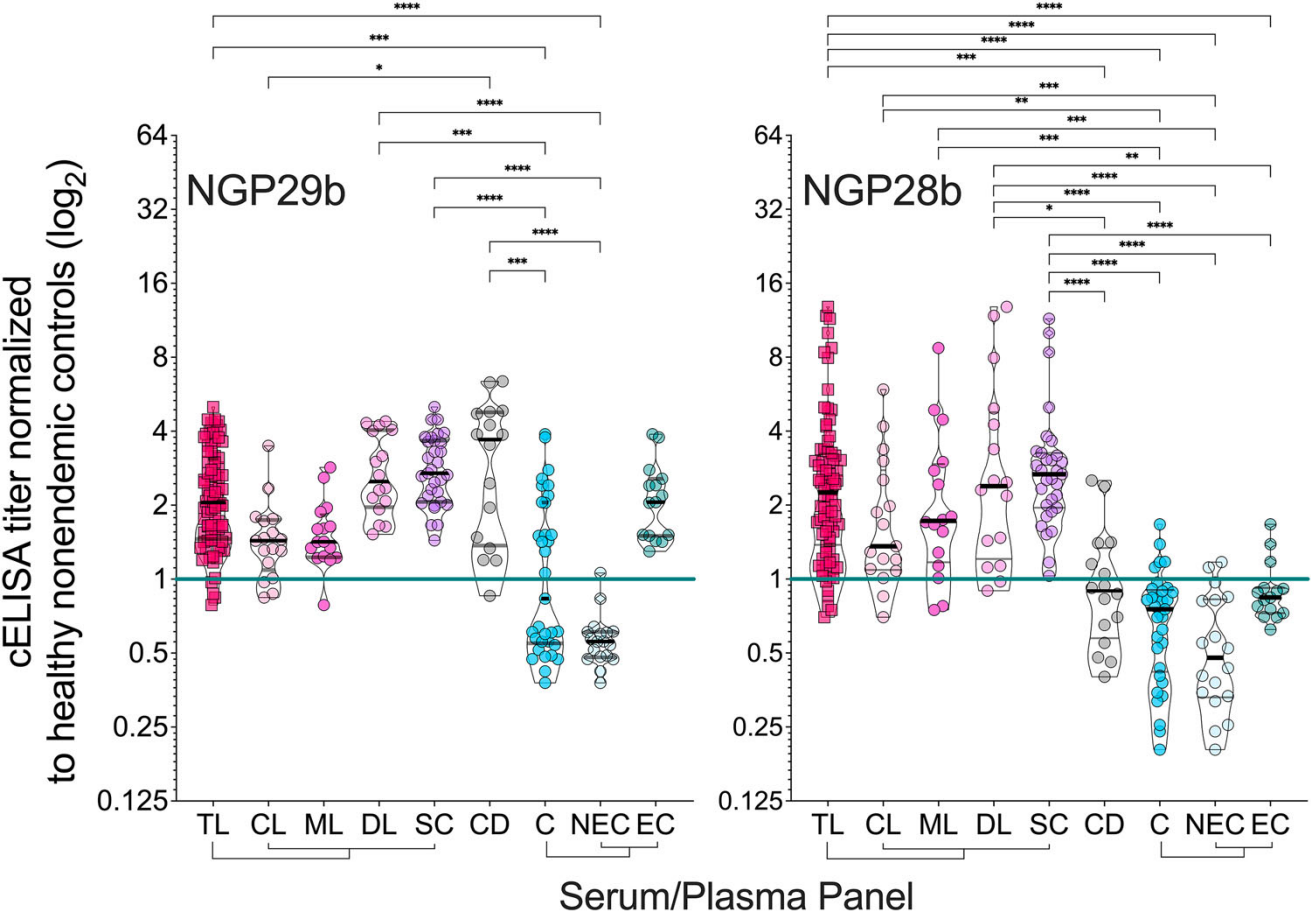
Normalized IgG response of sera from patients with tegumentary leishmaniasis (TL) to *L. major* type-2 GIPL-1-derived NGP29b and GIPL-3-derived NGP28b. cELISA immunoassays were performed using NGPs at 5 ng/well and serum samples (1:800 dilution) from all TL samples (n = 80), with different clinical forms (CL; n = 17; ML n = 16; DL, n = 16; and SC, n = 31) plotted separately; Chagas disease (CD, n = 16); and all non-TL, seemingly healthy controls (NEC + EC; n = 33), also plotted separately (EC, n = 15; and NEC, n = 18). Each point represents the mean of triplicate relative luminescence units (RLU) values normalized to NEC serum pools. The cutoff value (cELISA titer = 1.000), calculated as described in Materials and Methods, is indicated by the continuous green line. Data are represented as violin plots (truncated) of individual points, with median (thick black line) and interquartile range (dotted black lines) values indicated. *p < 0.05, **p < 0.01, ****p < 0.0001, Kruskal Wallis followed by Dunn’s multiple comparison tests. Statistically non-significant differences between serum groups are not shown.

Next, we evaluated individual sera (at 1:800 dilution) of patients representing the full TL clinical spectrum, CD patients, and endemic (non-TL) and nonendemic healthy controls. Based on previous results (Figure 2), we selected NGP29b and NGP28b as antigens for these assays. Due to the low reactivity of CL or CD serum pools to NGP30b, this antigen was not further pursued. Despite the diversity in clinical presentations, sera from patients across all clinical forms of TL reacted strongly to NGP29b and NGP28b (Figure 3). We established an initial cELISA titer cutoff (C*_i_*) of 1.000, determined in each microplate assay by using a pool of seemingly healthy nonendemic control sera (NEC, n = 15) in sextuplicate, as described in Material and Methods. NGP29b diagnosed 76/80 (sensitivity = 95.0%) of all TL patients as positive, being 14/17 (sensitivity = 82.4%) of CL, 15/16 (sensitivity = 93.8%) of ML, and 16/16 (sensitivity = 100%) of DL patients. (Figure 3, left panel; Table 2 and Table S1). On the other hand, NGP28b diagnosed as positive 74/80 (sensitivity = 92.5%) of total TL patients, being 15/17 (sensitivity = 88.2%) of CL patients, and 14/16 (sensitivity = 87.5%) of both ML and DL patients. For CD patients, NGP29b exhibited a 93.8% sensitivity (15/16), whereas NGP28b showed a 37.5% sensitivity value (6/16 individuals). In fact, CD patients and total controls (C = EC + NEC) exhibited a nonsignificant difference in the titers of anti-NGP28b antibodies (Figure 3, right panel; Table 3 and Table S1). Both antigens, however, detected as positive 100% of SC individuals, who do not present active ulcers but exhibit a positive LST response, an indicator of exposure to *Leishmania* spp. [48].

**Table 2.** Sensitivity, specificity, and other diagnostic parameters of type-2 GIPL-1-based NGP29b, in the comparison of different TL clinical forms vs. endemic and nonendemic controls.

Parameter	TL Clinical Forms vs. Endemic and Nonendemic Controls ^a^					
	TL (n = 80)	CL (n = 17)	ML (n = 16)	DL (n = 16)	SC (n = 31)	CD (n = 16)
*Original Values (%)^b^*						
Sensitivity	95.0	82.4	93.8	100.0	100.0	93.8
Specificity	48.5	48.5	48.5	48.5	48.5	48.5
FPR	51.5	51.5	51.5	51.5	51.5	51.5
PPV	80.0	45.2	46.9	48.5	64.6	46.9
NPV	81.4	84.2	94.1	100.0	100.0	94.1

^a^ Controls: endemic (EC) (n = 15) plus healthy nonendemic controls (NEC) (n = 18) individuals.

^b^ Values calculated based on the initial cutoff value (C*i*; titer = 1.000) (Figure 3A), as described in Material and Methods. Sensitivity = true positive (TP)/TP + false negative (FN). Specificity = true negative (TN)/TN + false positive (FP). False-positive rate (FPR)    100 − specificity. Positive predictive value (PPV)    TP/TP + FP. Negative predictive value (NPV)    TN/TN + FN.

**Table 3.** Sensitivity, specificity, and other diagnostic parameters of type-2 GIPL-3-based NGP28b, in the comparison of different TL clinical forms vs. endemic and nonendemic controls.

Parameter	TL Clinical Forms vs. Endemic and Nonendemic Controls ^a^					
	TL (n = 80)	CL (n = 17)	ML (n = 16)	DL (n = 16)	SC (n = 31)	CD (n = 16)
***Original values (%)^b^***						
Sensitivity	92.5	88.2	87.5	87.5	100.0	37.5
Specificity	84.9	84.9	84.9	84.9	84.9	84.9
FPR	15.2	15.2	15.2	15.2	15.2	15.2
PPV	93.7	75.0	73.7	73.7	86.1	54.6
NPV	82.4	93.3	93.3	93.3	100.0	73.7
***Post-TG-ROC Analysis (%)^c^***						
Sensitivity	92.5	88.2	87.5	93.8	93.6	37.5
Specificity	84.9	84.9	84.9	84.9	97.0	84.9
FPR	15.2	15.2	15.2	15.2	3.0	15.2
PPV	93.7	75.0	73.7	75.0	85.3	54.6
NPV	82.4	93.3	93.3	96.6	93.3	73.7

^a^ Controls: endemic (EC) (n = 15) plus healthy nonendemic controls (NEC) (n = 18) individuals.

^b^ Values calculated based on the initial cutoff value (Ci; titer = 1.000) (Figure 3B), as described in Material and Methods.

^c^ Values calculated based on the TG-ROC analysis (See Supplementary Figure 2).

We have also evaluated both NGPs for specificity by comparing sera from the TL forms caused by *L. braziliensis* with control (EC and NEC) sera. NGP29b exhibited a low specificity of 48.5% when we evaluated sera from all TL clinical forms studied and CD (Table 2). This result was due to 100% of EC sera (n = 15) being diagnosed as false-positive by NGP29b (Figure 3, left panel). Conversely, NGP28b exhibited 84.9% specificity comparing sera from total TL forms or individual CL, ML, DL, or SC form vs. EC and NEC controls (Table 3). NGP28b also successfully discriminated patients of all TL clinical forms from CD patients, with 92.5% sensitivity, 62.5% specificity, and AUC = 0.8684, indicating a strong discriminatory power (Table S2 and Figure S1).

**Figure 4.**
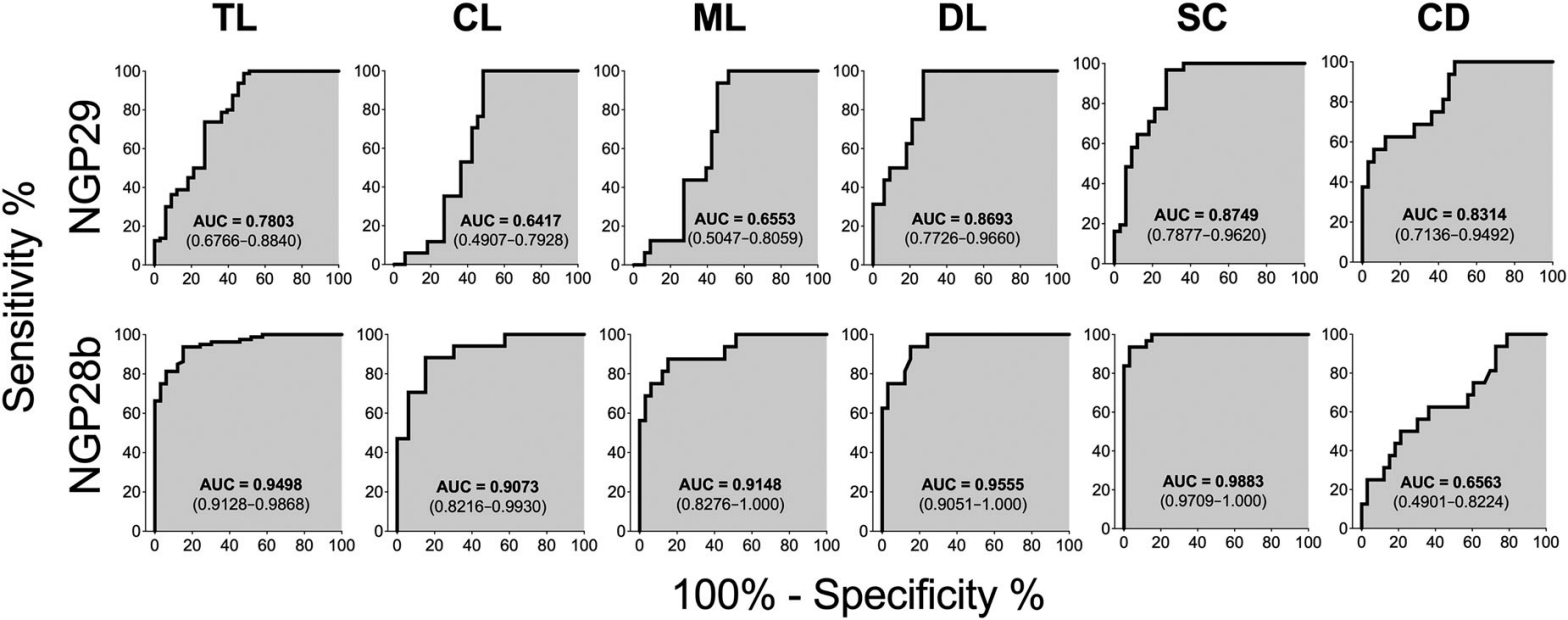
Receiver-operating characteristic (ROC) curves for NGP29b and NGP28b comparing the reactivity of sera from total TL patients or CL, ML, DL, SC, or CD patients versus control sera from endemic (EC) and nonendemic (NEC) individuals, using cELISA titers normalized to NECs. The area under the curve (AUC) is indicated in the gray area, and 95% confidence interval (CI) values are indicated in parentheses.

To further compare the capacity of NGP29b and NGP28b to discriminate sera from TL patients and SC individuals from EC and NEC sera, we performed ROC analysis using cELISA titers normalized to NEC serum pools. The AUC values of the ROC curves for the reactivity of NGP29b and NGP28b, respectively, with sera from total TL (0.7803 and 0.9498), CL (0.6417 and 0.9073), ML (0.6553 and 0.9148), DL (0.8693 and 0.9555), SC (0.8749 and 0.9883), and CD (0.8314 and 0.6563) patients confirmed the higher sensitivity and specificity of NGP28b compared to NGP29b across the different TL forms studied (Figure 4).

There is an urgent need for new serological diagnostic BMKs that could detect the broad spectrum of clinical presentations in TL, especially for surveillance during the pre-clinical phase or reactivation of disease. In this context, high sensitivity is preferred over high specificity for a new potential biomarker for TL. To that end, we then performed a two-graph ROC (TG-ROC) analysis of NGP28b by plotting the ROC data for sensitivity (Se) and specificity (Sp) as a function of the cELISA titer that defines the original cutoff (C*i*) value of 1.000, to fine-tune the analysis through cutoff adjustment [49]. For DL, the adjustment of the initial titer cutoff value (C*_i_*) of 1.000 to 0.9735 gave a higher sensitivity (93.8% from 87.5%), while maintaining the same specificity of 84.9%. For SC, although the adjustment from 1.000 to 1.454 of the titer cutoff value resulted in a lower sensitivity (93.6% from 100%), it significantly increased the specificity from 84.9% to 97%. The balance of cutoff values of sensitivity and specificity for total TL, CL, and ML diagnosis could not be significantly improved; therefore, we maintained the original C*_i_* of 1.000 (Table 3 and Figure S2).

**Figure 5.**
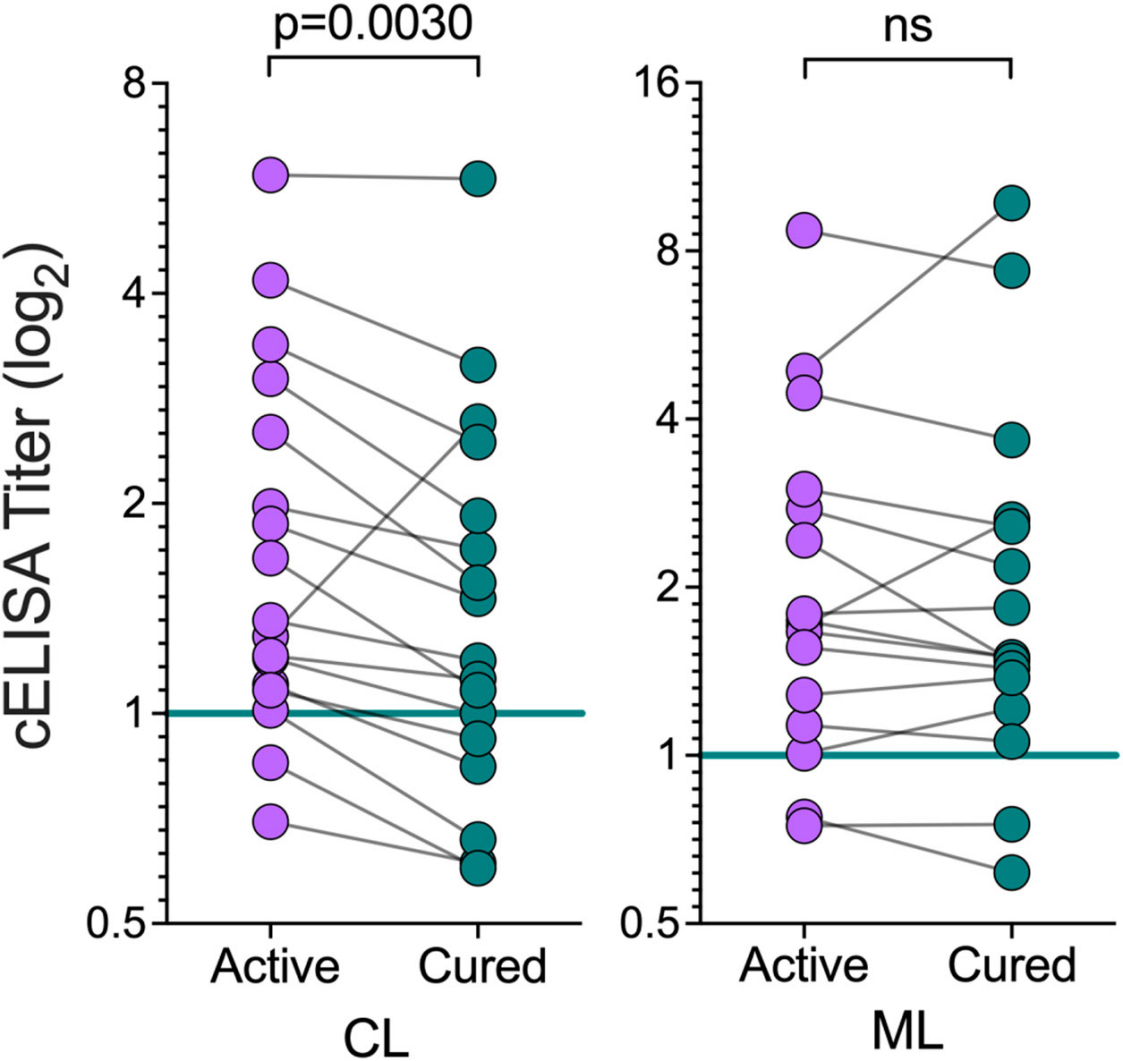
Seroreactivity to NGP28b of TL patients, before and after treatment. Sera from CL (n = 17) or ML (n = 16) patients, obtained before and 90 days after standard Sb^v^ treatment, were probed by cELISA with NGP28b (5 ng/well). Each point represents the mean of triplicate RLU values normalized to a pool of sera from seemingly healthy NEC individuals. *p* Values were calculated using Wilcoxon matched-pairs test. Significance level: *p *< 0.05.

Titers of anti-*Leishmania* IgG antibodies are known to decrease after successful chemotherapy of CL and ML [50,51]. Possible explanations are related to decreased circulating antigen and/or modulation of the immune response following parasite elimination [52]. Therefore, we also evaluated whether NGP28b-based cELISA could be used for monitoring the cure of CL and ML patients, with matched samples of patients before treatment (active disease) and 90 days after the onset of treatment (cured). Sera from cured CL patients exhibited significantly lower titers of anti-NGP28b (*p *= 0.003, Wilcoxon matched-pairs test) compared to serum samples from the same individuals with active disease. By contrast, overall, ML patients exhibited non-significant differences in the reactivity to NGP28b pre- and posttreatment (Figure 5). Of note, CL patients reached clinical cure significantly faster (45.1 ± 18.6 days) compared to ML patients (59.6 ± 17.7 days; *p* = 0.0294, CL vs. ML) (Table 1), which probably contributed to the lower titers of anti-NGP28b antibodies in the serum of cured CL patients (Figure 5). Collectively, our results showed that serology to NGP28b, an *L. major* type-2 GIPL-3-based NGP is applicable for the serodiagnosis of different clinical forms of TL caused by *L. braziliensis*, especially of asymptomatic SC forms. Moreover, NGP28b-based cELISA has the potential to be used as a BMK to monitor clinical cure following chemotherapy in CL patients.

## Discussion

The cell surface of all *Leishmania* species thus far studied is covered by a dense coat of glycosylphosphatidylinositol (GPI)-anchored glycoconjugates, containing or not a polypeptide chain. Among those that lack protein, GIPLs and LPG are the most abundant and studied GPI-anchored glycoconjugates, particularly those from Old-World *Leishmania* species (e.g., *L. major*, *L. donovani, L. tropica*, *L. aethiopica*). We have recently shown that synthetic NGPs containing two similar α-Gal glycotopes, Galα1,3Gal*f*β-BSA (NGP27b) and Galα1,3Gal*f*β1,3Manα-BSA (NGP30b), based on *L. major* type-2 GIPL-3, were highly antigenic and able to discriminate Old-World CL caused by *L. major* from that caused by *L. tropica* [24]. A previous study by Avila *et al.* has demonstrated that α-Gal-containing glycolipids purified from *L. braziliensis* promastigotes and comigrating with *L. major* type-2 GIPL-2 and GIPL-3 were highly antigenic for sera from patients with New-World or American TL caused by *L. braziliensis* [53]. Thus far, the detailed structure of the *L. braziliensis* GIPLs remain elusive. However, a preliminary structural analysis by Assis *et al.* indicates that *L. braziliensis* GIPLs are rich in galactose residues and could be similar to type-2 GIPLs of *L. major* [54]*,* as previously proposed by Avila and colleagues [53]*.* These studies made us to hypothesize that *L. major* type-2 GIPLs could be useful as diagnostic BMKs for different clinical forms of American TL.

As proof of concept, here we employed the reversed immunoglycomics approach [24], a bottom-up strategy that combines the chemical synthesis of potential glycotopes and conjugation to a carrier protein to generate NGPs, and probe them for antigenicity in serological immunoassays with patients’ sera. To this end, using sera from patients with different clinical forms of TL caused by *L. braziliensis*, we evaluated one terminal-β-Gal*f*-bearing NGP (NGP29b, Gal*f*β1,3Manα-BSA) and two terminal-α-Gal-bearing NGPs (NGP30b, Galα1,3Gal*f*β1,3Manα-BSA; and NGP28b, Galα1,6Galα1,3Gal*f*β-BSA), based on *L. major* type-2 GIPL-1, -2, and -3, respectively. Glycotopes containing α-Gal*p* and/or β-Gal*f* are abundant among trypanosomatids such as *T*. *cruzi* and some species of *Leishmania*, and are highly immunogenic to humans [30,43,44,55], making them potentially suitable for the purpose of specific and differential serodiagnosis of these diseases. Previous studies using New-World *Leishmania* species showed the potential of anti-α-Gal serological diagnosis in CL, ML, and diffuse cutaneous leishmaniasis (DCL) caused by *L. mexicana* [19], and in CL caused by *L. braziliensis* [19,21]. Here, we showed by cELISA that type-2 GIPL-3-based NGP28b (Galα1,6Galα1,3Gal*f*β-BSA) was the most reactive NGP to CL sera, in a dose-dependent and specific manner, as well as with TL sera from patients with other clinical forms. Moreover, NGP28b exhibited low cross-reactivity to CD and EC sera, indicating a strong discriminatory power. On the other hand, although highly reactive to sera from the same TL cohort, NGP29b (Gal*f*β1,3Manα-BSA) was also highly cross-reactive to sera from CD patients and EC individuals, but not with sera from NEC individuals. This result entirely agrees with a recent study showing that CD patients have very high levels of anti-β-Gal*f* IgG antibodies against Gal*f*β1,3Manα-BSA (NGP29b) and Gal*f*β1,3Manα1,2[Gal*f*β1,3]Manα-BSA (NGP32b) [32]. We cannot exclude the possibility of the EC individuals being infected by other infectious agents (e.g., fungi, bacteria, and/or parasites) that could elicit anti-β-Gal*f* IgG antibodies that could strongly recognize NGP29b. The high immunoreactivity of NGP29b to CD and its lack of specificity for TL vs. EC represents an issue for the use of this NGP as a diagnostic tool in rural areas of Bahia, where *T. cruzi* vectors still occur in domestic and peridomestic environments [56,57]. Thus, in areas where mixed CD and TL infections might occur, cross-reactivity to NGP29b could result in false-positive outcomes. Conversely, we showed that seroreactivity against NGP28b successfully discriminated TL from CD sera, with 92.5% sensitivity and 62.5% specificity (AUC = 0.8684) (Table S2 and Fig. S1), indicating a strong discriminatory power of this antigen. Obviously, a higher specificity would be desirable; thus, further improvements in that regard will be the focus of our future studies.

Several antigens have been proposed for a potential use in the serodiagnosis of leishmaniasis, replacing crude *Leishmania* spp. antigens [13–15]. Here we observed that serologic reactivity to NGP28b was higher in TL patients with either clinical disease or subclinical form. Subclinical *L. braziliensis* infection is characterized by the presence of a positive LST result in otherwise healthy subjects [48]. The LST is a measure of the cellular immune response that is determined after intradermal injection of leishmanial antigens. The ensuing delayed-type hypersensitivity response is evaluated 48 hours later. A serology-based assay, such as cELISA using synthetic NGP28b, would overcome this hurdle, accelerating the diagnosis of subclinical *L. braziliensis* infection. A positive seroreactivity to NGP28b among subclinical individuals suggests the possibility of identifying infected (asymptomatic) individuals before the development of disease, that is, allowing for diagnosis prior to the appearance of clinical manifestations. In visceral leishmaniasis caused by *L. donovani*, anti-rK39 immunoassays (ELISA and dipstick tests) were used to predict disease development in contacts of VL patients [58]. By means of a prospective study, authors reported a 44% predictive value for disease development in the three months following seroevaluation, and 57% probability in six months thereafter. Given the occurrence of post-kala-azar dermal leishmaniasis (PKDL) in *L. donovani* infection and the probable reservoir role of asymptomatic individuals, identification of asymptomatic carriers represents an important advance in disease control. Similar advantages are expected in the case of TL caused by *L. braziliensis*, especially given the possibility of occurrence of severe ML or DL.

The level of IgM anti-α-Gal antibodies in CL, caused by *L. mexicana* or *L. braziliensis,* specific to the Galα1,3Man glycotope expressed on these parasite phospholipids increases with the progression of disease [53]. Their levels are expected to be higher during active disease and decrease considerably after the decrease of the oligosaccharide stimulus provided by the parasite, suggesting that antibodies against α-Gal glycotopes could be useful for the early assessment of chemotherapeutic interventions in CL. In CL, sera obtained from individuals with active infection and post-cure recognize various α-Gal glycotopes, with different connectivity and secondary and tertiary epitopes linked to hydrophobic (lipid or protein) scaffold, on purified or synthetic molecules, indicating that numerous distinct pools of anti-α-Gal antibodies with different specificities and cross-reactivities might exist in these patients [19,21–23]. Earlier, comparisons of IgG levels to NGPs did not change drastically pre- and posttreatment in patients with CL caused by *L. major* [31]. Authors proposed that this was related to an accelerated recovery (lesion epithelization) time-frame (<1 month). Additionally, anti-*α*-Gal IgG remained high up to two years following initial detection, again in *L. major*-infected and cured individuals, suggesting longevity of anti-α-Gal B-cell clones specific to *L. major* [22]. In our setting, cured CL and ML patients still showed high IgG response to the Galα1,6Galα1,3Gal*f*β glycotope on NGP28b 90 days posttreatment with Sb^v^. However, a significant decrease in anti-α-Gal levels was observed for most cured CL patients, indicating that anti-NGP28b response could be a potential BMK for the presence of active CL. In our cohort, the time-to-heal period of ML patients was significantly longer compared to CL, suggesting that circulating/residual antigens might sustain the humoral response elevated, despite reepithelialization. As observed in treated adult CD patients [59,60], however, a much longer treatment follow-up period would be necessary to confirm whether or not a decreasing anti-α-Gal antibody trend in CL and ML patients might correlate with the current cure criterion (reepithelialization) [33].

Our results show that a cELISA with an NGP based on a type-2 *L. major* GIPL-3 containing terminal Galα1,6Galα1,3Gal*f*β glycan is applicable for the serodiagnosis of TL caused by *L. braziliensis*, ranging from subclinical (SC) infection to severe disseminated (DL) disease, with high sensitivity and specificity. Moreover, in CL, the humoral immune response to NGP28b decreases with clinical cure indicating that this serology-based immunoassay could be potentially useful for monitoring response to chemotherapy. It will be interesting to determine, in the future, whether such *Leishmania*-specific anti-α-Gal antibodies persist in CL patients and, if so, whether this persistence, prospectively, may be a biomarker for the development of mucosal disease.

In summary, our results indicate that NGP28b, containing the Galα1,6Galα1,3Gal*f*β glycotope found on type-2 *L. major* GIPL-3, could be employed for the: (1) differential diagnosis of American tegumentary leishmaniasis from Chagas disease, which remains a major confounding factor in Brazil and in many other endemic areas of Latin America, where an epidemiologic overlap between the two infections exists; (2) diagnosis and epidemiological surveys of the subclinical form of TL, since no other biomarker has thus far been described and the nonspecific LST is the only laboratory tool available; (3) transmission surveillance of TL in endemic and nonendemic areas; and (4) follow-up of treated CL patients, after performing a prospective, well-controlled, and long-term clinical trial with a large cohort that guarantees enough statistical power for a robust statistical analysis. This study is, above all, the proof of concept that reversed immunoglycomics using synthetic *Leishmania*-based glycans coupled to a carrier protein could be a viable approach for the development of molecular tools for a more accurate diagnosis and chemotherapy follow-up of distinct clinical forms of TL.

## Supplementary Material

Supplemental Material
