# Supplementary material for: Serodiagnosis and therapeutic monitoring of New-World tegumentary leishmaniasis using synthetic type-2 glycoinositolphospholipid-based neoglycoproteins
Source: Emerg Microbes Infect. 2022 Sep 21;11(1):2147–59. doi: 10.1080/22221751.2022.2114852 (PMC9518598; doi:10.1080/22221751.2022.2114852)
Supplement: Supplemental Material [file TEMI_A_2114852_SM2038.pdf]

Serodiagnosis and therapeutic monitoring of New-World tegumentary leishmaniasis using synthetic type-2 glycoinositolphospholipid-based neoglycoproteins

Sayonara M. Viana[1,a], Alba L. Montoya[2,b], Augusto M. Carvalho[1,4], Brunele S. de Mendonça[1], Susana Portillo[3,c], Janet J. Olivas[3,d], Nasim H. Karimi[3,e], Igor L. Estevao[3], Uriel Ortega-Rodriguez[3,f], Edgar M. Carvalho[1,4], Walderez O. Dutra[4,5], Rosa A. Maldonaldo[3], Katja Michael[2], Camila I. de Oliveira[1,4,*,¶], and Igor C. Almeida[3,*,¶]

[1] Instituto Gonçalo Moniz, Fundação Oswaldo Cruz (FIOCRUZ), Salvador, BA, Brazil.

[2] Department of Chemistry and Biochemistry, Border Biomedical Research Center, The University of Texas at El Paso, El Paso, Texas, U.S.A.

[3] Department of Biological Sciences, Border Biomedical Research Center, The University of Texas at El Paso, El Paso, Texas, U.S.A.

[4] Instituto Nacional de Ciência e Tecnologia de Doenças Tropicais, Salvador, BA, Brazil.

[5] Departamento de Morfologia, Instituto de Ciências Biológicas, Universidade Federal de Minas Gerais (UFMG), Belo Horizonte, MG, Brazil

Supplemental Material

Table S1. Seroreactivity to NGP29b (GIPL-1-based) and NGP28b (GIPL-3-based) of different clinical forms of TL.

Disease / Clinical Form / Control	*n*	NGP29b		NGP28b	
		Positive	Negative	Positive	Negative
		Original Values [a]			
Tegumentary leishmaniasis	80	76	4	74	6
CL	17	14	3	15	2
ML	16	15	1	14	2
DL	16	16	0	14	2
SC	31	31	0	31	0
Chagas disease	16	15	1	6	10
Endemic control	15	15	0	3	12
		Post-TG-ROC Analysis Values [b]			
Tegumentary leishmaniasis	80	76	4	74	6
CL	17	14	3	15	2
ML	16	15	1	14	2
DL	16	16	0	15	1
SC	31	31	0	29	2
Chagas disease	16	15	1	6	10
Endemic control	15	15	0	3	12

[a] Values calculated based on the initial cutoff value (C*i*; titer = 1.000) (Figure 3), as described in Material and Methods.

[b] Values calculated based on the TG-ROC analysis (see Supplementary Figure 1).

Table S2. Sensitivity, specificity, and other diagnostic parameters of type-2 GIPL-3-based NGP28b, in the comparison of different TL clinical forms vs. Chagas disease.

**Parameter**	**TL Infection Forms vs. Chagas Disease**				
	**TL (n = 80)**	**CL (n = 17)**	**ML (n = 16)**	**DL (n = 16)**	**SC (n = 31)**
	Original Values (%) [a]				
Sensitivity	92.5	88.2	87.5	87.5	100.0
Specificity	62.5	62.5	62.5	62.5	62.5
FPR	37.5	37.5	37.5	37.5	37.5
PPV	92.5	71.4	70.0	70.0	83.8
NPV	92.5	83.3	83.3	83.3	100.0

[a] Values calculated based on the initial cutoff value (C_i ; titer = 1.000) (Figure 3B), as described in Material and Methods. Sensitivity = true positive (TP)/TP + false negative (FN). Specificity = true negative (TN)/TN + false positive (FP). False-positive rate (FPR) = 100 − specificity. Positive predictive value (PPV) = TP/TP + FP. Negative predictive value (NPV) = TN/TN + FN.

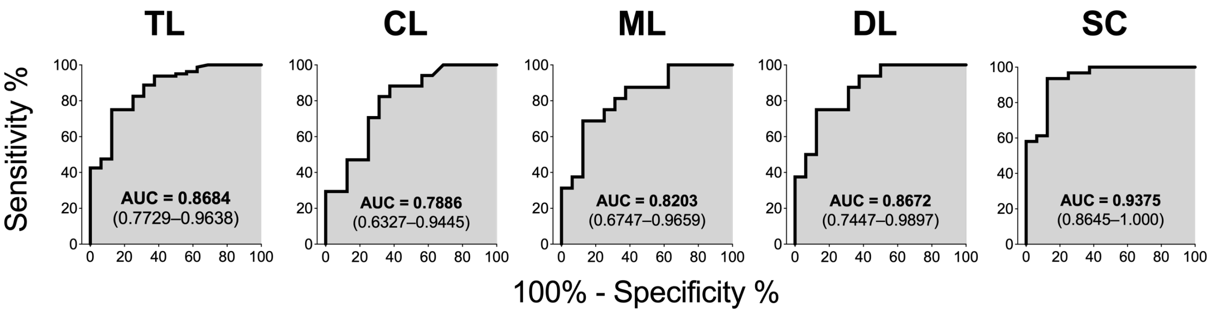

Supplementary Figure 1. Receiver-operating characteristic (ROC) curves for NGP28b comparing the reactivity of sera from total TL patients, or CL, ML, DL, or SC patients versus CD patients, using cELISA titers normalized to NECs. The AUC is indicated in the gray area, and 95% CI values are indicated in parentheses.

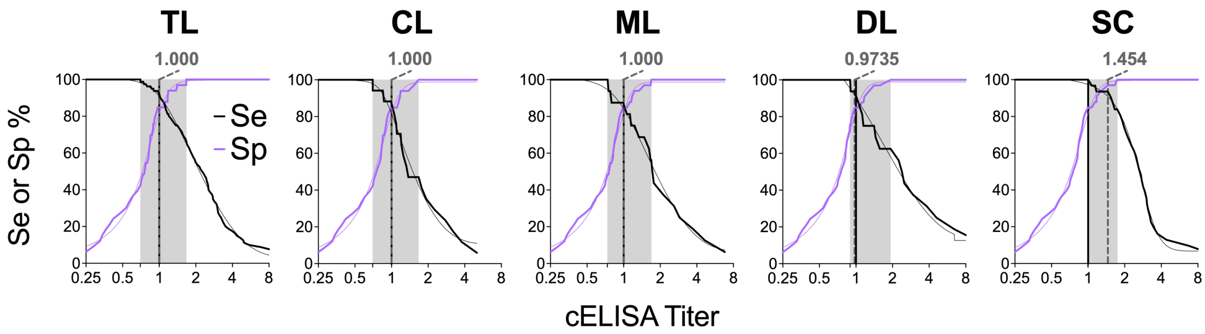

Supplementary Figure 2. Two-graph (TG)-ROC curve analysis was performed for by plotting the ROC data for sensitivity (Se, black lines) and specificity (Sp, purple lines) against NGP28b for all TL patients or CL, ML, DL, or SC patients versus EC+NEC individuals. The Se and Sp raw data points are represented as thick lines, whereas the best-fitted data are indicated as smooth fine lines. Shaded area indicates the cELISA titer interval where Se or Sp could reach 100%. Vertical black line, initial cELISA titer cutoff value (C_i=1.000); vertical dotted gray line, adjusted cELISA titer value (C_a, gray number on top) for the comparison of TL, CL, ML, DL, or SC patients versus controls (EC+NEC). Note that, for TL, CL, and ML patients, no C_a value was generated and the C_i=1.000 remained as the cutoff.
